# Factors Affecting Combination Trial Success (FACTS): Investigator Survey Results on Early-Phase Combination Trials

**DOI:** 10.3389/fmed.2019.00122

**Published:** 2019-06-04

**Authors:** Channing J. Paller, Erich P. Huang, Thomas Luechtefeld, Holly A. Massett, Christopher C. Williams, Jinxiu Zhao, Amy E. Gravell, Tami Tamashiro, Steven A. Reeves, Gary L. Rosner, Michael A. Carducci, Lawrence Rubinstein, S. Percy Ivy

**Affiliations:** ^1^Department of Oncology, Sidney Kimmel Comprehensive Cancer Center, Johns Hopkins School of Medicine, Baltimore, MD, United States; ^2^Biometrics Research Program, Division of Cancer Treatment and Diagnosis, National Cancer Institute, Rockville, MD, United States; ^3^Environmental Health Sciences, Johns Hopkins Bloomberg School of Public Health, Baltimore, MD, United States; ^4^Cancer Therapy Evaluation Program, Division of Cancer Treatment and Diagnosis, National Cancer Institute, Rockville, MD, United States; ^5^Clinical Trials Information Management Services, Emmes Corporation, Rockville, MD, United States; ^6^Coordinating Center for Clinical Trials, National Cancer Institute, Rockville, MD, United States; ^7^Division of Biostatistics and Bioinformatics, Sidney Kimmel Comprehensive Cancer Center, Johns Hopkins School of Medicine, Baltimore, MD, United States

**Keywords:** clinical trials, combination therapy, regulatory approval, early phase, trial design, drug combinations, phase 1 trials

## Abstract

Experimental therapeutic oncology agents are often combined to circumvent tumor resistance to individual agents. However, most combination trials fail to demonstrate sufficient safety and efficacy to advance to a later phase. This study collected survey data on phase 1 combination therapy trials identified from ClinicalTrials.gov between January 1, 2003 and November 30, 2017 to assess trial design and the progress of combinations toward regulatory approval. Online surveys (*N* = 289, 23 questions total) were emailed to Principal Investigators (PIs) of early-phase National Cancer Institute and/or industry trials; 263 emails (91%) were received and 113 surveys completed (43%). Among phase 1 combination trials, 24.9% (95%CI: 15.3%, 34.4%) progressed to phase 2 or further; 18.7% (95%CI: 5.90%, 31.4%) progressed to phase 3 or regulatory approval; and 12.4% (95%CI: 0.00%, 25.5%) achieved regulatory approval. Observations of “clinical promise” in phase 1 combination studies were associated with higher rates of advancement past each milestone toward regulatory approval (cumulative OR = 11.9; *p* = 0.0002). Phase 1 combination study designs were concordant with Clinical Trial Design Task Force (CTD-TF) Recommendations 79.6% of the time (95%CI: 72.2%, 87.1%). Most discordances occurred where no plausible pharmacokinetic or pharmacodynamic interactions were expected. Investigator-defined “clinical promise” of a combination is associated with progress toward regulatory approval. Although concordance between study designs of phase 1 combination trials and CTD-TF Recommendations was relatively high, it may be beneficial to raise awareness about the best study design to use when no plausible pharmacokinetic or pharmacodynamic interactions are expected.

## Introduction

Recent advances in genomic sequencing (1), molecular characterization of cancers and companion biomarkers (2), immune system knowledge (3), and other areas of research are uncovering new cancer therapies. These novel therapies are reshaping the field of cancer medicine and increasingly being evaluated in combination with other novel drugs as well as with approved treatments (4) in an effort to circumvent tumor resistance to individual agents, enhance synergy, and employ dual pathway inhibition. Combination trials now account for more than 25% of clinical trials in oncology, and trials supported by the National Institutes of Health (NIH) are significantly more likely to use drug combinations than those supported by industry (5). Trials involving combination of agents pose distinct challenges, including design of clinical trials that provide informative results, selection of agents with acceptable toxicity and improved efficacy and logistical and regulatory challenges. Phase I trials are the initial step in combination regimen clinical evaluation. This article presents an assessment of phase 1 combination trials in ClinicalTrials.gov between January 1, 2003 and November 30, 2017 to determine the proportion that achieved regulatory approval, and factors associated with success.

To date, most drug combination trials fail to demonstrate sufficient safety and efficacy to advance to later phases of development (6). The design and conduct of early-phase combination trials present specific challenges, such as determining which agents to combine, choosing an appropriate dose and schedule (including which agent to escalate), and addressing drug-drug interactions and overlapping toxicities (7, 8). Furthermore, supportive measures that may effectively treat chemotherapy-related toxicities are insufficient in dealing with toxicities brought on by molecularly targeted agents, including rashes and elevated liver transaminases (9). Molecularly targeted agents usually require continuous dosing until disease progression, and thus are associated with toxicities that may not have been observed during the dose-limiting toxicity (DLT) assessment period, are more difficult to manage, and are exacerbated in combination therapy trials (9).

**Figure 1:**
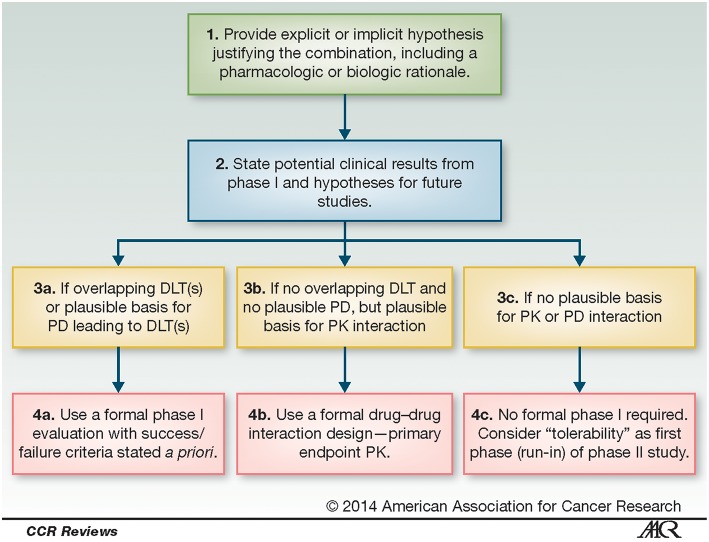
Process for determining clinical trial design of phase 1 combinations. These recommendations for the design of phase 1 combination clinical trials, from the Clinical Trial Design Task Force of the NCI Investigational Drug Steering Committee, address expected overlapping dose-limiting toxicities (DLTs) or plausible pharmacokinetic (PK) and pharmacodynamic (PD) interactions, as well as rationale and potential clinical results. Reproduced from Paller et al. (10) no permissions required.

Given the increasing importance of combination regimens and the challenges associated with their development, the National Cancer Institute (NCI) Investigational Drug Steering Committee appointed a Clinical Trial Design Task Force (CTD-TF) to develop pragmatic clinical guidelines for the design of phase 1 combination clinical trials that were published in 2014 (10). The guidelines, shown in Figure 1 recommend investigators use a biologic or pharmacologic rationale supported by clinical, preclinical and/or other evidence to justify the combination, describe next steps in development of the combination and potential clinical results, and then take into account overlapping DLTs and potential pharmacodynamic (PD) and pharmacokinetic (PK) interactions in order to select the most effective trial design.

The Task Force members agreed on a set of five factors that need to be considered in early-phase combination clinical trial design: (i) therapeutic effect (11, 12); (ii) mechanism of action and related PD markers (13–15); (iii) toxicity (e.g., non-overlapping dose limiting toxicities, chronic administration toxicity) (16); (iv) PK such as drug-drug interactions in which one drug may alter the metabolism of another and reduce or enhance its anticancer effect (17); and (v) dose schedule (e.g., low-dose continuous administration vs. high-dose intermittent administration) (18–20).

Publication bias results in limited data from negative studies available in journals, making literature reviews to characterize differences between positive and negative trials problematic (21). This study tests the hypothesis that a survey (Factors Affecting Combination Trial Success–FACTS) can be used to improve understanding of phase 1 trial design decisions. Specifically, the FACTS survey aimed to (i) assess proportions of combinations achieving each milestone toward regulatory approval, (ii) identify factors associated with these proportions, and (iii) assess the extent to which phase 1 trials were concordant with the CTD-TF guidelines. Because the CTD-TF guidelines were designed to help translational researchers improve the probability that a combination will advance toward regulatory approval, concordance with those guidelines may be a marker for predicting regulatory approval of the combination.

We relied on a survey for this work, despite the limitations of this approach, because theinformation we sought was only rarely included and nearly always incomplete in the manuscripts that were published and, of course, was not available at all when clinical trials were undertaken but no manuscript had been published. Thus, critically important patterns of clinical trial design decision-making was available only in the memories of the PIs. The Investigational Drug Steering Committee of NCI published new design guidelines in 2014 with the goal of improving the rate of success of early phase clinical trials in successfully move new treatments forward toward regulatory approval. We used the PI survey, then, to try to measure progress toward implementation of the new guidelines as a step toward accelerating adoption of those guidelines.

## Methods

### Participants

Survey participants were principal investigators (PIs) of early-phase cancer treatment trials that evaluated combinations of experimental therapeutic agents. To identify PIs eligible for this study, we conducted a search of the ClinicalTrials.gov database in September 2015 to identify cancer intervention clinical trials listed as phase 1, 1b, or 1/2 that evaluated combinations of two or more therapeutic agents (*N* = 389) in both solid tumors and hematologic malignancies. The therapeutic agents included molecularly targeted agents, immune-oncology drugs, and antibody drug conjugates as well as chemotherapies. The list of participants was updated with additional queries to ClinicalTrials.gov through November 2017. Contact information was available for 289 trials led by 243 PIs (36 PIs were responsible for multiple trials, range 2–6.), a majority were Cancer Therapy Evaluation Program (CTEP) investigators from the Experimental Therapeutics Clinical Trials Network (ETCTN; *n* = 138) under the NCI. The protocol (***FACTS_R02PAPP01)*** was approved by the Cancer Therapy Evaluation Program (CTEP) on June 6, 2017.

### Survey

A 23-question online survey was developed to collect information on trial design decisions made by the PI and the progress the combination made toward regulatory approval. Three key content areas were assessed within the survey: (i) biomarker decisions (types of biomarkers in the study, whether clinical data was used for rationale, and the presence of primary/secondary biomarker objectives); (ii) phase 1 combination decisions (trial design type, preclinical factors supporting the combination, pre-defined criteria used to determine success/failure, expected interactions, and results of the phase 1 trial, including further investigation warranted, secondary endpoints met, and results published); and (iii) status of combination progression (current status of the phase 2/phase 3 of combination, results of the phase 2/phase 3 trial, whether the phase 2/phase 3 met secondary endpoints, whether the phase 2/phase 3 results were published, and whether regulatory approval of the combination was granted). Additional questions asked about whether the trial was investigator-initiated, the trial's funding source, and PI familiarity with the 2014 CTD-TF recommendations. In-depth phone interviews were conducted with five PIs prior to survey dissemination to review and revise the survey draft questions to ensure clarity and comprehension of the questions.

### Endpoints

#### Milestone Achievements in Clinical Trial Development

**Figure 2:**
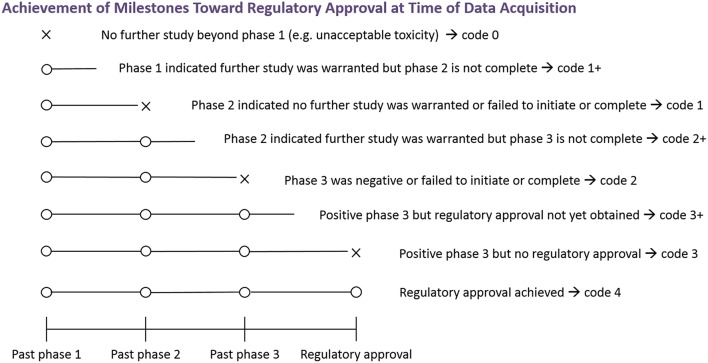
Achievement of milestones toward regulatory approval at time of data acquisition. The figure depicts the endpoint relating to achievement of milestones toward regulatory approval. An X indicates failure at that phase, and an O indicates successful advancement at that phase. An open line indicates that the highest milestone ultimately achieved is not known at the time of data acquisition. To obtain the numerical coding, add the number of Os. Studies of some combinations may be in progress at the time of data acquisition and the highest milestone ultimately achieved is not currently known. Outcomes of such combinations are indicated by a “+”.

The endpoint for this analysis was the number of clinical trial milestones each combination successfully achieved (i.e., further investigation beyond phase 1, further investigation beyond phase 2, positive phase 3 results, and regulatory approval; see Figure 2). Note that the investigation of some combinations was still in progress at the time of data acquisition (e.g., the phase 2 trial was positive, but the phase 3 trial was not yet initiated). For these combinations, the outcome is right-censored, as the highest milestone ultimately achieved was unknown, but was greater than or equal to the one achieved at data acquisition. This scenario was indicated by a “+” (e.g., if a phase 3 trial was ongoing, the endpoint was 2+).

#### Concordance Between CTD-TF Recommendations and Phase 1 Study Design

Concordance meant any of the following:

- Overlapping DLTs or plausible PD leading to DLTs were expected and a formal phase 1 evaluation with pre-defined success criteria was used.
- No overlapping DLTs and no plausible PD interactions were expected, but plausible PK interactions were, and a drug-drug interaction design with a PK primary endpoint was used.
- No plausible PD or PK interactions were expected, and no formal phase 1 study was performed.

### Procedure

An online survey platform was developed by Insilica Corporation at NCITrialPub.org to automatically generate email invitations to PIs and collect and manage the data. Survey links were created for each eligible trial (*N* = 289) and emailed to PIs from July-December 2017 in batches of 30 every week by the Emmes Corporation, an NCI contractor. Emails were re-sent to non-responders after 10 business days for a maximum of 5 reminders.

## Statistical Methodology

Maximum likelihood estimation was used to estimate the probabilities of achieving each milestone; the likelihood function and how right-censoring was handled are detailed in the Supplementary Methods. Likelihood ratio tests were used to assess the associations between individual study characteristics and the probabilities of achieving each milestone, with the Benjamini-Hochberg procedure (22) used to adjust for multiple testing. Multivariate models of these probabilities given the study characteristics were constructed using logistic regression subject to Elastic Net constraints (see Supplementary Methods).

The proportion of combinations in which the phase 1 trial study design and CTD-TF Recommendations were concordant was estimated with 95% confidence intervals. A chi-square test was used to assess whether the expected interactions and DLTs were independent of the study design used. A Mann-Whitney *U* Test was used to assess the association between familiarity of the PI with CTD-TF Recommendations and concordance of the phase 1 trial study design with CTD-TF Recommendations.

## Results

**Figure 3:**
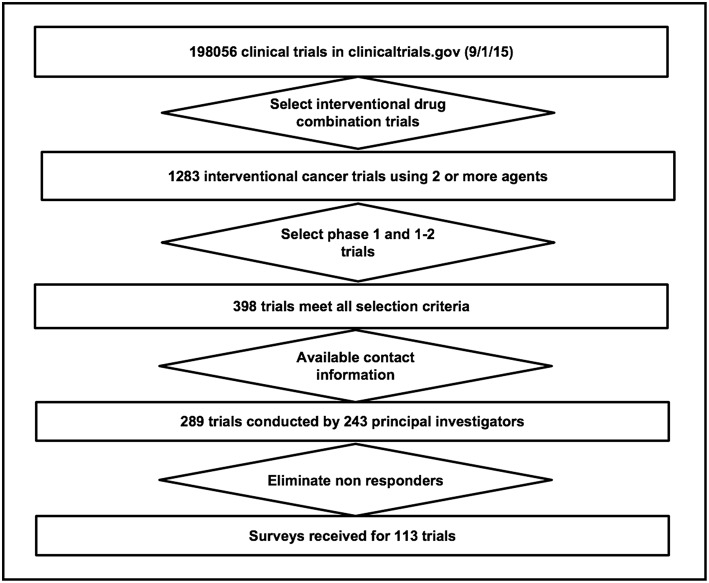
CONSORT diagram for this study.

The survey was dispatched to 289 PIs between July and December 2017. Delivery was successful for 263 surveys, and valid responses were received for 113 (39%) trials (Figure 3). A data verification in which two coders reviewed the literature on 10% of the combinations and answered the survey questions independently showed 99% agreement between the publications and the survey responses.

### Probabilities of Advancement Past Each Milestone Toward Regulatory Approval

Of the combinations, 39.8% (45/113; 95% CI: 30.8%, 48.8%) advanced beyond phase 1. The estimate for the proportion advancing beyond phase 2 was 24.9% (95% CI: 15.3%, 34.4%), and 15 of the 113 combinations in the data had achieved this milestone by the time of data acquisition. Note that the estimate of the proportion advancing beyond a milestone may not be equal to the proportion of combinations in the data achieving the milestone at the time of data acquisition due to the right-censoring. The former takes into account that combinations may achieve additional milestones in the future.

The estimate for the proportion for which the phase 3 trial was positive was 18.7% (95% CI: 5.90%, 31.4%); three of the 113 combinations were associated with a positive phase 3 trial by the time of data acquisition. The estimate of the proportion achieving regulatory approval was 12.4% (95% CI: 0.00%, 25.5%), with two of the 113 combinations achieving regulatory approval by the time of data acquisition. These results are shown in Table 1.

**Table 1:** Summary statistics for the advancement of combinations past the various milestones in clinical trial development.

**Milestone**	**Probability of achieving milestone (with 95% confidence interval)**	**Probability of achieving milestone given successful achievement of all preceding ones (with 95% confidence interval)**	**Number of combinations known to have achieved milestone**	**Number of combinations known to have failed milestone given successful achievement of all preceding ones**
Past phase 1	39.8% (30.8%, 48.8%)	39.8% (30.8%, 48.8%)	45	68
Past phase 2	24.9% (15.3%, 34.4%)	62.5% (43.1%, 81.9%)	15	9
Past phase 3	18.7% (5.90%, 31.4%)	75.0% (32.6%, 100%)	3	1
Regulatory approval	12.4% (0.00%, 25.5%)	66.7% (13.3%, 100%)	2	1

### Concordance Between Phase 1 Study Design and CTD-TF Recommendations

Table 2 compares expected DLTs and PK and PD interactions with the type of phase 1 study design used, and shows concordance between CTD-TF Recommendations and phase 1 study design was observed in 79.6% of the interactions (90 out of 113; 95% CI: 72.2%, 87.1%). However, formal phase 1 designs with pre-determined success criteria were used in 110 of the 113 surveyed trials, including in all 20 trials in which the CTD-TF would not have recommended using this design. The *p*-value of the test of independence between expected DLTs and PK and PD interactions vs. the type of phase 1 study used was 0.956. Investigators whose designs were in concordance with the CTD-TF Recommendations reported greater familiarity with the guidelines than PIs in non-concordant studies (19% vs. 3% reporting they were “very familiar”) (Table 3). The Mann-Whitney U Test of the degree of familiarity with CTD-TF Recommendations vs. concordance of phase 1 study designs with CTD-TF Recommendations indicated little evidence of association between these two variables (*p* = 0.304). Because 108 out of 113 of the surveyed trials were submitted to ClinicalTrials.gov before the Recommendations were published in August 2014 (10), low concordance was to be expected. However, the data in this study provide a baseline against which to measure improvement in concordance over time.

**Table 2:** Expected dose-limiting toxicities (DLTs) and pharmacodynamic (PD) or pharmacokinetic (PK) interactions vs. the type of phase 1 study design in surveyed trials.

**Expected DLTs and PD or PK interactions**	**Formal phase 1 evaluation with pre-determined success criteria**	**Drug-drug interaction design with PK primary endpoint**	**No formal phase 1**
Overlapping DLTs or plausible PD leading to DLTs	90	3	0
No overlapping DLTs, no plausible PD, plausible PK	1	0	0
No plausible PK or PD interaction	19	0	0

*Cells along the diagonal indicate those in which the study design was concordant with Clinical Trial Design Task Force (CTD-TF) Recommendations (shown in ***Figure 1***)*.

**Table 3:** Familiarity of the Principal Investigator (PI) with Clinical Trial Design Task Force (CTD-TF) recommendations vs. concordance between the study design used in the phase 1 trial and CTD-TF recommendations.

**PI familiarity with CTD-TF Recommendations**	**Design of phase 1 study not concordant with CTD-TF Recommendations**	**Design of phase 1 study concordant with CTD-TF Recommendations**
Not familiar	9	27
Somewhat familiar	11	44
Very familiar	3	19

### Phase 1 Study Characteristics Associated With Advancement Toward Regulatory Approval

Summary statistics for the phase 1 combination survey results are provided in Table 4. At the α = 0.05 level, the data provided evidence of a significant association between clinical promise in the phase 1 trial (i.e., evidence of sufficient activity, e.g., decrease in tumor size or FDG uptake or prolonged progression-free or overall survival, at tolerable levels of toxicity to move forward with a registration-directed investigation) and advancement toward regulatory approval (*p* = 0.0002). Clinical promise was associated with higher probabilities of achieving each of the milestones toward regulatory approval (Table 5): 66.7% of combinations with observed clinical promise progressed past phase 1, whereas only 23.9% of those without observed clinical promise did; 40% of combinations with observed clinical promise progressed past phase 2, whereas only 16% of those without observed clinical promise did; 40% of combinations with observed clinical promise progressed past phase 3, whereas only 10.6% of those without observed clinical promise did; 40% of combinations with observed clinical promise achieved regulatory approval, whereas only 5.32% of those without observed clinical promise did; and 53 of the 113 combinations required the observation of clinical promise in their success criteria. Clinical promise was observed in about half of the 53 trials for which showing preliminary evidence of activity was a criterion for the success of the phase 1 trial. The 60 trials that did not explicitly require a demonstration of evidence still showed clinical promise 25% of the time, and 68% of these trials still advanced past phase 1 when clinical promise was observed.

**Table 4:** Summary statistics for the phase 1 study characteristics.

**Phase 1 study characteristic**	**Distribution in data**	**Notes**
Establishing safe and tolerable dose or schedule is criterion for phase 1 success	TRUE: 100/113 (88.5%) FALSE: 13/113 (11.5%)	Investigators were asked to indicate whether any of these pre-defined criteria were used to decide whether to move forward to phase 2 or cease development
Establishing optimal dose or schedule is criterion for phase 1 success	TRUE: 30/113 (26.5%) FALSE: 83/113 (73.5%)	
Determining drug administration sequence is criterion for phase 1 success	TRUE: 1/113 (0.88%) FALSE: 112/113 (99.1%)	
Showing PD effect is criterion for phase 1 success	TRUE: 14/113 (12.4%) FALSE: 99/113 (87.6%)	
Characterizing PK is criterion for phase 1 success	TRUE: 24/113 (21.2%) FALSE: 89/113 (78.8%)	
Showing preliminary evidence of activity is criterion for phase 1 success	TRUE: 53/113 (46.9%) FALSE: 60/113 (53.1%)	
Study design used for phase 1	3 + 3: 98/113 (86.7%) Adaptive model-based: 8/113 (7.08%) Drug-drug interaction: 3/113 (2.65%) Other: 4/113 (3.54%)	
Clinical data used for pharmacological or biological rationale for study of the combination	TRUE: 75/113 (66.4%) FALSE: 38/113 (33.6%)	
Safe and tolerable dose or schedule established in phase 1	TRUE: 79/113 (69.9%) FALSE: 34/113 (30.1%)	Investigators were asked to indicate whether any of these results were observed in their phase 1 trial. Clinical promise refers to evidence of activity at tolerable levels of toxicity. Other results encompass any other results not covered by the other options
Optimal dose or schedule established in phase 1	TRUE: 23/113 (20.4%) FALSE: 90/113 (79.6%)	
Drug administration sequence determined in phase 1	TRUE: 2/113 (1.77%) FALSE: 111/113 (98.2%)	
Pre-planned PD effect shown in phase 1	TRUE: 7/113 (6.19%) FALSE: 106 (93.8%)	
Other PD effects observed in phase 1	TRUE: 10/113 (8.85%) FALSE: 103/113 (91.2%)	
PK observed in phase 1	TRUE: 29/113 (25.7%) FALSE: 84/113 (74.3%)	
Clinical promise observed in phase 1	TRUE: 42/113 (37.2%) FALSE: 71/113 (62.8%)	
Other results observed in phase 1	TRUE: 19/113 (16.8%) FALSE: 94/113 (83.2%)	
Rationale for study of combination based on *in vitro* evidence of greater activity of the combination	TRUE: 82/113 (72.6%) FALSE: 31/113 (27.4%)	Investigators were asked to indicate whether any of these were rationales for the study of their combination
Rationale for study of combination based on *in vivo* evidence of greater activity of the combination	TRUE: 43/113 (38.1%) FALSE: 70/113 (61.9%)	
Rationale for study of combination based on lack of overlapping toxicities	TRUE: 31/113 (27.4%) FALSE: 82/113 (72.6%)	
PD biomarker-driven objectives included in phase 1 trial	TRUE: 29/113 (25.7%) FALSE: 84/113 (74.3%)	Investigators were asked to indicate whether the objectives of their phase 1 study involved any of these biomarker types. Pharmacodynamic (PD) biomarkers relate to the effect relative to a targeted defect or pathway. Predictive biomarkers explain differences in effect between multiple classes of treatment. Prognostic biomarkers indicate the likely outcome of the patient under standard therapy. Response biomarkers are used to assess changes in the underlying physiology following treatment
Predictive biomarker-driven objectives included in phase 1 trial	TRUE: 11/113 (9.73%) FALSE: 102/113 (90.3%)	
Prognostic biomarker-driven objectives included in phase 1 trial	TRUE: 2/113 (1.77%) FALSE: 111/113 (98.2%)	
Response biomarker-driven objectives included in phase 1 trial	TRUE: 17/113 (15.0%) FALSE: 96/113 (85.0%)	
Exploratory biomarker-driven objectives included in phase 1 trial	TRUE: 26/113 (23.0%) FALSE: 87/113 (77.0%)	
Other biomarker-driven objectives included in phase 1 trial	TRUE: 0/113 (0%) FALSE: 113/113 (100%)	
PD interactions expected	TRUE: 11/113 (9.73%) FALSE: 102/113 (90.3%)	Investigators were asked to indicate whether any of these potentially negative drug-drug interactions may be observed with their combination
PK interactions expected	TRUE: 12/113 (10.6%) FALSE: 101/113 (89.4%)	
Adverse events expected	TRUE: 54/113 (47.8%) FALSE: 59/113 (52.2%)	
Overlapping dose-limiting toxicities expected	TRUE: 42/113 (37.2%) FALSE: 71/113 (62.8%)	
Concerns about possible interactions tested for in phase 1 trial	TRUE: 69/113 (61.1%) FALSE: 44/113 (38.9%)	

*Some characteristics (e.g., criteria for phase 1 study success, phase 1 study results, and phase 1 study objectives) were recoded as a series of binary variables associated with each possible choice. Study characteristics for which the observed values were imbalanced (i.e., at least 90% of cases were equal to one value) were omitted from this analysis*.

**Table 5:** Probabilities of successfully achieving each milestone toward regulatory approval.

**Phase 1 study characteristic**	**Past phase 1**	**Past phase 2**	**Past phase 3**	**Regulatory approval**	**Likelihood ratio test *p*-value**
Clinical promise observed in phase 1	TRUE: 66.7% FALSE: 23.9%	TRUE: 40.0% FALSE: 16.0%	TRUE: 40.0% FALSE: 10.6%	TRUE: 40.0% FALSE: 5.32%	**0.0002**
Other results observed in phase 1	TRUE: 10.5% FALSE: 45.7%	TRUE: 10.5% FALSE: 27.8%	TRUE: 10.5% FALSE: 18.6%	TRUE: 0.00% FALSE: 18.6%	0.005
Response biomarker-driven objectives included in phase 1 trial	TRUE: 58.8% FALSE: 36.5%	TRUE: 49.0% FALSE: 20.3%	TRUE: 0.00% FALSE: 20.3%	TRUE: 0.00% FALSE: 13.5%	0.060
Characterizing PK is criterion for phase 1 success	TRUE: 29.2% FALSE: 42.7%	TRUE: 0.00% FALSE: 30.5%	TRUE: 0.00% FALSE: 22.9%	TRUE: 0.00% FALSE: 15.2%	0.087
Safe and tolerable dose or schedule established in phase 1	TRUE: 44.3% FALSE: 29.4%	TRUE: 25.6% FALSE: 23.5%	TRUE: 0.00% FALSE: 23.5%	TRUE: 0.00% FALSE: 15.7%	0.106
Clinical data used for pharmacological or biological rationale for study of the combination	TRUE: 36.0% FALSE: 47.4%	TRUE: 22.2% FALSE: 30.1%	TRUE: 11.1% FALSE: 30.1%	TRUE: 0.00% FALSE: 30.1%	0.141
PK interactions expected	TRUE: 8.33% FALSE: 43.6%	TRUE: 0.00% FALSE: 27.2%	TRUE: 0.00% FALSE: 20.4%	TRUE: 0.00% FALSE: 13.6%	0.152
Showing PD effect is criterion for phase 1 success	TRUE: 21.4% FALSE: 42.4%	TRUE: 0.00% FALSE: 28.9%	TRUE: 0.00% FALSE: 21.7%	TRUE: 0.00% FALSE: 14.5%	0.155
Optimal dose or schedule established in phase 1	TRUE: 52.2% FALSE: 36.7%	TRUE: 39.1% FALSE: 22.0%	TRUE: 39.1% FALSE: 0.00%	TRUE: 26.1% FALSE: 0.00%	0.156
Concerns about possible interactions tested for in phase 1 trial	TRUE: 34.8% FALSE: 47.7%	TRUE: 20.3% FALSE: 31.8%	TRUE: 20.3% FALSE: 21.2%	TRUE: 0.00% FALSE: 21.2%	0.162
Rationale for study of combination based on *in vitro* evidence of greater activity of the combination	TRUE: 36.6% FALSE: 48.4%	TRUE: 20.9% FALSE: 33.9%	TRUE: 0.00% FALSE: 33.9%	TRUE: 0.00% FALSE: 22.5%	0.184
Rationale for study of combination based on lack of overlapping toxicities	TRUE: 38.7% FALSE: 40.2%	TRUE: 11.1% FALSE: 30.8%	TRUE: 0.00% FALSE: 23.1%	TRUE: 0.00% FALSE: 15.4%	0.303
Showing preliminary evidence of activity is criterion for phase 1 success	TRUE: 39.6% FALSE: 40.0%	TRUE: 15.8% FALSE: 31.4%	TRUE: 0.00% FALSE: 23.6%	TRUE: 0.00% FALSE: 15.7%	0.441
Adverse events expected	TRUE: 40.7% FALSE: 38.6%	TRUE: 27.2% FALSE: 22.7%	TRUE: 27.2% FALSE: 11.4%	TRUE: 13.6% FALSE: 11.4%	0.560
Rationale for study of combination based on *in vivo* evidence of greater activity of the combination	TRUE: 41.9% FALSE: 38.6%	TRUE: 25.1% FALSE: 24.8%	TRUE: 12.6% FALSE: 24.8%	TRUE: 12.6% FALSE: 12.4%	0.568
Establishing safe and tolerable dose or schedule is criterion for phase 1 success	TRUE: 38.0% FALSE: 53.8%	TRUE: 23.5% FALSE: 35.9%	TRUE: 15.7% FALSE: 35.9%	TRUE: 7.84% FALSE: 35.9%	0.569
Study design used for phase 1	3 + 3: 39.8% Adaptive: 37.5% Drug-drug: 66.7% Other: 25.0%	3 + 3: 21.9% Adaptive: 37.5% Drug-drug: 66.7% Other: 25.0%	3 + 3: 14.6% Adaptive: 0.00% Drug-drug: 0.00% Other: 25.0%	3 + 3: 14.6% Adaptive: 0.00% Drug-drug: 0.00% Other: 0.00%	0.615
PD biomarker-driven objectives included in phase 1 trial	TRUE: 27.6% FALSE: 44.0%	TRUE: 18.4% FALSE: 26.9%	TRUE: 0.00% FALSE: 20.2%	TRUE: 0.00% FALSE: 13.5%	0.630
Exploratory biomarker-driven objectives included in phase 1 trial	TRUE: 34.6% FALSE: 41.4%	TRUE: 23.1% FALSE: 25.3%	TRUE: 23.1% FALSE: 16.9%	TRUE: 23.1% FALSE: 8.43%	0.704
Establishing optimal dose or schedule is criterion for phase 1 success	TRUE: 36.7% FALSE: 41.0%	TRUE: 18.3% FALSE: 26.1%	TRUE: 18.3% FALSE: 17.4%	TRUE: 18.3% FALSE: 8.69%	0.729
Overlapping dose-limiting toxicities expected	TRUE: 33.3% FALSE: 43.7%	TRUE: 16.7% FALSE: 29.1%	TRUE: 0.00% FALSE: 21.8%	TRUE: 0.00% FALSE: 14.6%	0.790
PK observed in phase 1	TRUE: 41.4% FALSE: 39.3%	TRUE: 16.6% FALSE: 26.9%	TRUE: 0.00% FALSE: 20.2%	TRUE: 0.00% FALSE: 13.4%	0.850

*P-values of the likelihood ratio test of univariate associations between phase 1 study characteristics and probability of achieving each milestone toward regulatory approval (arranged in order of ascending p-values), and the estimates of the probabilities of achieving each milestone given each characteristic. The p-values for statistically significant univariate associations after adjustment with Benjamini-Hochberg are indicated in boldface. Univariate associations were not assessed for characteristics that were overwhelmingly absent (i.e., absent in 95% or more of combinations)*.

Table 6 lists regression coefficient estimates for a multivariate model of progression toward regulatory approval given study characteristics. The regression coefficient estimates associated with observed clinical promise in the phase 1 study and inclusion of phase 1 biomarker-driven objectives were linked to higher probabilities of progressing past each clinical trial milestone. The regression coefficient estimates associated with the following characteristics were negative, indicating that they were linked to lower probabilities of progressing past each milestone: the rationale for the combination study was based on *in vitro* evidence of activity; results other than establishing safe, tolerable, or optimal doses, determining the sequence of drug administration, or observing pharmacokinetic or pharmacodynamic effects or clinical promise in the phase 1 trial; PK interactions were expected; PK was a pre-defined criterion for the success of the phase 1 study; and overlapping dose-limiting toxicities were expected of the combination.

**Table 6:** Regression coefficient estimates and permutation test *p*-values of the multivariate model of probability of achieving each milestone given the phase 1 study characteristics.

**Phase 1 study characteristic**	**Regression coefficient estimate**	**Permutation test *p*-value**
Clinical promise observed in phase 1	**1.690**	**6.00 × 10^−4^**
Rationale for study of combination based on *in vitro* evidence of greater activity of the combination	**−1.634**	**1.80 × 10^−3^**
Response biomarker-driven objectives included in phase 1 trial	**1.761**	**2.50 × 10^−3^**
Characterizing PK is criterion for phase 1 success	**−2.323**	**2.70 × 10^−3^**
Other results observed in phase 1	**−1.893**	**8.30 × 10^−3^**
PK interactions expected	**−1.602**	**0.012**
Overlapping dose-limiting toxicities expected	**−0.970**	**0.015**
Rationale for study of combination based on lack of overlapping toxicities	−0.908	0.021
3 + 3 design used for phase 1	−1.007	0.028
Clinical data used for pharmacological or biological rationale for study of the combination	−0.711	0.036
Adverse events expected	0.673	0.040
Rationale for study of combination based on *in vivo* evidence of greater activity of the combination	0.586	0.044
PK observed in phase 1	0.727	0.046
Optimal dose or schedule established in phase 1	0.772	0.055
Safe and tolerable dose or schedule established in phase 1	0.726	0.061
PD biomarker-driven objectives included in phase 1 trial	−0.352	0.089

*The p-values for statistically significant associations after adjustment with Benjamini-Hochberg are indicated in boldface. Characteristics not selected through forward stepwise regression were not included in this table*.

## Discussion

Observing clinical promise of a combination (e.g., sufficient activity at tolerable levels of toxicity to warrant moving forward with registration-based investigation) in a phase 1 trial is associated with progress toward regulatory approval. However, nearly one-quarter of phase I trials that did not report clinical promise from phase 1 still moved into a phase 2 study. We estimate that 12% of all combinations will ultimately achieve regulatory approval: While only 5% of combinations that do not report clinically promising results in phase 1 achieve regulatory approval, 40% of combinations that do report clinically promising phase 1 results achieve regulatory approval. Only 47% of surveyed trials referenced clinical promise as a requirement of success, but clinical promise was nonetheless observed in 25% of the trials that *did not* require the observation. In trials lacking the clinical promise requirement, clinical promise was still strongly associated with phase 1 success. The data may indicate that clinical promise should be closely examined in phase 1 trials especially given that clinical promise was observed in ~50% of the trials that require it, but only observed 25% of the time in trials that did not require it and strongly associated with success in both cases (64–68% of trials where clinical promise was observed past phase 1). Further, these data may suggest investigators consider foregoing phase 2 studies for combinations that show little phase 1 clinical promise.

Although concordance of phase 1 designs with the CTD-TF Recommendations occurred in 79.6% of the trials, formal phase 1 designs were used in 97% of trials, including in all 20 cases (18%) in which the CTD-TF would not have recommended this design. Thus, a large proportion of investigators employ formal phase 1 designs even when expected interactions indicate that formal phase 1 designs are not ideal (*p*-value of test of independence of expected interactions and design: 0.956). This high level of concordance between phase 1 designs and CTD-TF Recommendations occurred despite more than 95% of the trials being submitted to ClinicalTrials.gov before the Recommendations were published. Follow-up with trials designed after the Recommendations were published will be needed to determine the impact of the Recommendations on improving factors toward success. Because greater familiarity was associated with concordance with the CTD-TF guidelines, additional benefit may be gained by raising awareness of the best study design to use when no plausible pharmacokinetic or pharmacodynamic interactions are expected.

Even with a sample size of 113, evidence of signal was found in some trial design characteristics with regard to advancement toward regulatory approval. Observation of phase 1 clinical promise to move forward with registration-directed investigation was significantly associated with advancement past each milestone toward regulatory approval. In addition, evidence of association with advancement toward regulatory approval were observed for (i) biomarker-driven objectives included in phase 1 design, (ii) assessment of therapeutic pharmacokinetic levels in phase 1, and (iii) findings from phase 1 trials other than establishment of safe, tolerable, or optimal doses, determining the sequence of drug administration, or observation of pharmacokinetic or pharmacodynamic effects or clinical promise. These associations are consistent with reported causes of failure of oncology drugs in late-stage clinical development that demonstrated lack of a biomarker-driven strategy and failure to attain proof of concept (23). In addition, observation of any pharmacodynamic or pharmacokinetic interactions was associated with lower probabilities of achieving all subsequent milestones toward regulatory approval, a finding consistent with reports of overlapping toxicities as significant contributors to the failure of drug combinations to reach regulatory approval (4).

Extending this survey to more combinations, including those not evaluated in CTEP-sponsored trials, will not only improve power to detect associations between these design characteristics and advancement toward regulatory approval, but also allow development and evaluation of a predictor of whether a combination will achieve each of these milestones based on one or more of these trial characteristics. Such a predictor may help inform investigators and funding sources in determining which combinations to include in phase 1 trial design.

One major limitation of this study is that we excluded chemo-radiation combinations. Our rationale was that we were seeking consistency of endpoints. We anticipate follow-on research will include combinations with radiation. The potential for bias based on only 39% response rate is another limitation as is the potential for recall bias. Although we are asking investigators to report information going back many years, the investigators played pivotal roles in the design, execution, and manuscript preparation, so their recall may be stronger than respondents without that close association. In addition, our survey instrument linked the responding investigator to that investigator's publication on the trial to help them with accuracy of recall. Our initial focus on CTEP-funded trials provided consistent, complete information that provides a strong launch of the FACTS program. A more inclusive database of combination trials, with regular progress updates toward regulatory approval and additional curation of structured data on clinical trials, may help to automatically identify promising clinical trials and/or alert practitioners of potential problems in their trial design.

## Disclosure

The authors have also confirmed that this article is unique and not under consideration or published in any other publication, and that they have permission from rights holders to reproduce any copyrighted material.

## Author Contributions

CP and EH wrote the first draft of the manuscript. CP, EH, TL, HM, AG, and TT contributed to the writing of the manuscript. CP, EH, TL, HM, CW, JZ, AG, TT, SI, GR, MC, and LR agree with manuscript results and conclusions. CP, EH, TL, HM, CW, JZ, AG, TT, SR, GR, MC, LR, and SI jointly developed the structure and arguments for the paper. CP, EH, TL, HM, AG, GR, MC, LR, and SI made critical revisions and approved final version. All authors reviewed and approved of the final manuscript.

### Conflict of Interest Statement

The authors declare that the research was conducted in the absence of any commercial or financial relationships that could be construed as a potential conflict of interest. The handling editor declared a shared affiliation, though no other collaboration, with several of the authors CP, GR, and MC at time of review.

## References

[1] Torshizi AD Wang K Next-generation sequencing in drug development: target identification and genetically stratified clinical trials. Drug Discov Today. (2018) 23:1776–83. 10.1016/j.drudis.2018.05.015 29758342

[2] Smith AD Roda D Yap TA. Strategies for modern biomarker and drug development in oncology. J Hematol Oncol. (2014) 7:70. 10.1186/s13045-014-0070-8 25277503 PMC4189730

[3] Yuan J Hegde PS Clynes R Foukas PG Harari A Kleen TO. Novel technologies and emerging biomarkers for personalized cancer immunotherapy. J Immunother Cancer. (2016) 4:3. 10.1186/s40425-016-0107-3 26788324 PMC4717548

[4] Day D Siu LL. Approaches to modernize the combination drug development paradigm. Genome Med. (2016) 8:115. 10.1186/s13073-016-0369-x 27793177 PMC5084460

[5] Wu M Sirota M Butte AJ Chen B Characteristics of drug combination therapy in oncology by analyzing clinical trial data on ClinicalTrials.gov. Pac Symp Biocomput. (2014) 2015:68–79. PMC4361221 25592569

[6] Maitland ML Hudoba C Snider KL Ratain MJ. Analysis of the yield of phase II combination therapy trials in medical oncology. Clin Cancer Res. (2010) 16:5296–302. 10.1158/1078-0432.CCR-10-0669 20837695 PMC2970723

[7] Riviere MK Le Tourneau C Paoletti X Dubois F Zohar S. Designs of drug-combination phase I trials in oncology: a systematic review of the literature. Ann Oncol. (2015) 26:669–74. 10.1093/annonc/mdu516 25403591

[8] Cannistra SA. Challenges and pitfalls of combining targeted agents in phase I studies. J Clin Oncol. (2008) 26:3665–7. 10.1200/JCO.2008.17.2676 18669449

[9] Yap TA Rodon J. Development of molecularly driven targeted combination strategies. Oncologist. (2017) 22:1421–3. 10.1634/theoncologist.2017-0402 29038233 PMC5728037

[10] Paller CJ Bradbury PA Ivy SP Seymour L LoRusso PM Baker L. Design of phase I combination trials: recommendations of the Clinical Trial Design Task Force of the NCI Investigational Drug Steering Committee. Clin Cancer Res. (2014) 20:4210–7. 10.1158/1078-0432.CCR-14-0521 25125258 PMC4135521

[11] Cunningham D Humblet Y Siena S Khayat D Bleiberg H Santoro A. Cetuximab monotherapy and cetuximab plus irinotecan in irinotecan-refractory metastatic colorectal cancer. N Engl J Med. (2004) 351:337–45. 10.1056/NEJMoa033025 15269313

[12] Geyer CE Forster J Lindquist D Chan S Romieu CG Pienkowski T. Lapatinib plus capecitabine for HER2-positive advanced breast cancer. N Engl J Med. (2006) 355:2733–43. 10.1056/NEJMoa064320 17192538

[13] Tolcher AW Baird RD Patnaik A Moreno V Papadopoulos KP Garrett CR A phase I dose-escalation study of oral MK-2206 (allosteric AKT inhibitor) with oral selumetinib (AZD6244; MEK inhibitor) in patients with advanced or metastatic solid tumors. J Clin Oncol. (2011) 29(Suppl. 15):3004–3004. 10.1200/jco.2011.29.15_suppl.3004

[14] Baselga J Bradbury I Eidtmann H Di Cosimo S de Azambuja E Aura C. Lapatinib with trastuzumab for HER2-positive early breast cancer (NeoALTTO): a randomised, open-label, multicentre, phase 3 trial. Lancet. (2012) 379:633–40. 10.1016/S0140-6736(11)61847-3 22257673 PMC5705192

[15] Carver BS Chapinski C Wongvipat J Hieronymus H Chen Y Chandarlapaty S. Reciprocal feedback regulation of PI3K and androgen receptor signaling in PTEN-deficient prostate cancer. Cancer Cell. (2011) 19:575–86. 10.1016/j.ccr.2011.04.008 21575859 PMC3142785

[16] Kwak EL Clark JW Chabner B. Targeted agents: the rules of combination. Clin Cancer Res. (2007) 13(18 pt 1):5232–7. 10.1158/1078-0432.CCR-07-1385 17875749

[17] Stearns V Johnson MD Rae JM Morocho A Novielli A Bhargava P. Active tamoxifen metabolite plasma concentrations after coadministration of tamoxifen and the selective serotonin reuptake inhibitor paroxetine. J Natl Cancer Inst. (2003) 95:1758–64. 10.1093/jnci/djg108 14652237

[18] Seymour L Ivy SP Hilton J Dancey J Paller C Clinical Interactions with combinations of novel agents. At: International Congress on Targeted Anti-Cancer Therapies, Amsterdam. Ann Oncol. (2012) 23(Suppl. 1):i15–25, L6.4. 10.1093/annonc/mds017

[19] Hamberg P Ratain MJ Lesaffre E Verweij J. Dose-escalation models for combination phase I trials in oncology. Eur J Cancer. (2010) 46:2870–8. 10.1016/j.ejca.2010.07.002 20691584

[20] Luechtefeld T Maertens A McKim JM Hartung T Kleensang A Sa-Rocha V. Probabilistic hazard assessment for skin sensitization potency by dose-response modeling using feature elimination instead of quantitative structure-activity relationships. J Appl Toxicol. (2015) 35:1361–71. 10.1002/jat.3172 26046447 PMC4805435

[21] Song F Parekh-Bhurke S Hooper L Loke Y Ryder J Sutton A. Extent of publication bias in different categories of research cohorts: a meta-analysis of empirical studies. BMC Med Res Methodol. (2009) 9:79. 10.1186/1471-2288-9-79 19941636 PMC2789098

[22] Benjamini Y Hochberg Y Controlling the false discovery rate: a practical and powerful approach to multiple testing. J R Stat Soc Series B Methodol. (1995) 57:289–300. 10.1111/j.2517-6161.1995.tb02031.x

[23] Jardim DL Groves ES Breitfeld PP Kurzrock R. Factors associated with failure of oncology drugs in late-stage clinical development: a systematic review. Cancer Treat Rev. (2017) 52:12–21. 10.1016/j.ctrv.2016.10.009 27883925

