# Supplementary material for: Factors Affecting Combination Trial Success (FACTS): Investigator Survey Results on Early-Phase Combination Trials
Source: Front Med (Lausanne). 2019 Jun 4;6:122. doi: 10.3389/fmed.2019.00122 (PMC6558040; doi:10.3389/fmed.2019.00122)
Supplement: Supplementary file 1 [file Table_1.DOCX]

**Supplementary Methods**

**Model**

Let $y_{i}$ denote the number of milestones toward regulatory approval that the $i^{th}$ combination has achieved at the time of data acquisition and let $Y_{i}^{*}$ denote the number of milestones toward regulatory approval this combination will ultimately achieve. For some combinations, the highest milestone ultimately achieved is unknown at the time of data acquisition (e.g. the Phase 2 study of a combination may be in progress when the data are collected). This induces some right-censoring into the outcome; $Y_{i}^{*}$ is yet to be ascertained but is known to be greater than or equal to $y_{i}$. Let $z_{i}=I\left\{ Y_{i}^{*}=y_{i} \right\}$ be an indicator of whether the highest milestone that the $i^{th}$ study ultimately achieves is known at the time of data acquisition. Note that for this analysis, if $y_{i}=0$, then $z_{i}=1$ necessarily as failure to achieve any milestones is mutually exclusive of any of the other possible outcomes. Also, with $M$ milestones toward regulatory approval, if $y_{i}=M$, then $z_{i}=1$ necessarily also since achievement of the $M^{th}$ milestone is the highest possible outcome.

Note that due to the sequential nature of the milestones (i.e. if a study has successfully achieved the $y^{th}$ milestone, then it has also successfully achieved all lower milestones as well), i.e.

| $P\left( Y_{i}^{*}\geq y_{i} \right)=P\left( Y_{i}^{*}\geq y_{i}, Y_{i}^{*}\geq y_{i}-1, \ldots, Y_{i}^{*}\geq1 \right)$ $=P\left( Y_{i}^{*}\geq y_{i} \vert Y_{i}^{*}\geq y_{i}-1 \right)P\left( Y_{i}^{*}\geq y_{i}-1 \vert Y_{i}^{*}\geq y_{i}-2 \right)\times\ldots\times P\left( Y_{i}^{*}\geq1 \right)$ $=\prod_{m=0}^{y_{i}} \theta_{m}$ | (A1) |
| --- | --- |

where $\theta_{m}=P\left( Y_{i}^{*}\geq m | Y_{i}^{*}\geq m-1, \ldots, Y_{i}^{*}\geq1 \right)=P\left( Y_{i}^{*}\geq m | Y_{i}^{*}\geq m-1 \right)$ is the conditional probability that a study successfully achieves the $m^{th}$ milestone given that it has also achieved the first $m-1$milestones and $\theta_{1}=P(Y_{i}^{*}\geq1)$ is the probability that a study at least achieves the lowest milestone. To facilitate notation later, set $\theta_{0}=1$; also note that $\theta_{m}=0$ for $m>M$. Therefore, the probability that a study ultimately achieves the first $y_{i}$ milestones is

| $P\left( Y_{i}^{*}=y_{i} \right)=P\left( Y_{i}^{*}\geq y_{i} \right)-P\left( Y_{i}^{*}\geq y_{i}-1 \right)$ $=\prod_{m=0}^{y_{i}} \theta_{m}-\prod_{m=0}^{y_{i}+1} \theta_{m}$  $=\left( 1-\theta_{y_{i}+1} \right)\prod_{m=0}^{y_{i}} \theta_{m}$ | (A2) |
| --- | --- |

**Maximum Likelihood Estimation**

The likelihood function is then

| $L\left( \theta\right)= \prod_{i=1}^{N} \left[ P\left( Y_{i}^{*}=y_{i} \right) \right]^{z_{i}}\left[ P\left( Y_{i}^{*}\geq y_{i} \right) \right]^{1-z_{i}}$ $=\prod_{i=1}^{N} \left[ \left( 1- \theta_{y_{i}+1} \right)\prod_{m=0}^{y_{i}} \theta_{m} \right]^{z_{i}}\left[ \prod_{m=0}^{y_{i}} \theta_{m} \right]^{1-z_{i}}$ $=\prod_{i=1}^{N} \left[ \left( 1-\theta_{y_{i}+1} \right)^{z_{i}}\prod_{m=0}^{y_{i}} \theta_{m} \right]$ | (A3) |
| --- | --- |

where $\theta=\left( \theta_{1},\ldots,\theta_{M} \right)^{T}$. Note that

| $\prod_{m=0}^{y_{i}} \theta_{m}=\prod_{m=1}^{M} \theta_{m}^{I\left\{ y_{i}\geq m \right\}}$ | (A4) |
| --- | --- |
| $1-\theta_{y_{i}+1}=\prod_{m=1}^{M} \left( 1-\theta_{m} \right)^{I\left\{ y_{i}=m-1 \right\}}$ | (A5) |

Therefore,

| $L\left( \theta\right)=\prod_{i=1}^{N} \prod_{m=1}^{M} \left( 1-\theta_{m} \right)^{I\left\{ y_{i}=m-1 \right\}z_{i}}\theta_{m}^{I\left\{ y_{i}\geq m \right\}}$ | (A6) |
| --- | --- |

The maximum likelihood estimators for estimators for $\theta_{m}$ for $m=1, \ldots, M$ are

| $\hat{\theta}_{m}=\frac{\sum_{i=1}^{N} I\left\{ y_{i}\geq m \right\}}{\sum_{i=1}^{N} I\left\{ y_{i}\geq m \right\}+\sum_{i=1}^{N} I\left\{ y_{i}=m-1 \right\}z_{i}}$ | (A7) |
| --- | --- |

The maximum likelihood estimators for the probabilities of achieving each milestone is then

| $\hat{P}\left( Y_{i}^{*}\geq y \right)=\prod_{m=0}^{y} \hat{\theta}_{m}$ | (A8) |
| --- | --- |

The covariance matrix for the maximum likelihood estimators (i.e. the inverse of the observed information matrix), denoted $\Sigma\left( \theta\right)$, is a diagonal matrix whose $m^{th}$ diagonal entry is

| $\left[ \Sigma\left( \hat{\theta} \right) \right]_{mm}=\frac{\hat{\theta}_{m}\left( 1-\hat{\theta}_{m} \right)}{\sum_{i=1}^{N} I\left\{ y_{i}\geq m \right\}+\sum_{i=1}^{N} I\left\{ y_{i}=m-1 \right\}z_{i}}$ | (A9) |
| --- | --- |

The covariance matrix for the maximum likelihood estimators of $P\left( Y_{i}^{*}\geq y \right)$ can be obtained through the Delta Method. Let

| $p\left( \theta\right)=\left( P\left( Y_{i}^{*}\geq1 \right), P\left( Y_{i}^{*}\geq2 \right), \ldots, P\left( Y_{i}^{*}\geq M \right) \right)^{T}$ $=\left( \theta_{1}, \theta_{1}\theta_{2},\ldots, \prod_{m=1}^{M} \theta_{m} \right)^{T}$ | (A10) |
| --- | --- |

Then the Jacobian $\left( \theta\right)$ is an $M \times M$ matrix whose $y^{th}$ row and $m^{th}$ column equals

| $\left[ J\left( \theta\right) \right]_{ym}=\frac{\partial}{\partial\theta_{m}}P(Y\geq y)$ $=\left\{ \begin{matrix} 0 & \mathrm{if} m\geq y \\ \frac{\prod_{k=1}^{y} \theta_{k}}{\theta_{m}} & \mathrm{otherwise} \end{matrix} \right.$ | (A11) |
| --- | --- |

Standard errors for $P\left( Y_{i}^{*}\geq y \right)$ are therefore

| $\hat{\mathrm{se}}\left[ \hat{P}\left( Y_{i}^{*}\geq y \right) \right]=\sqrt{\left[ J\left( \theta\right)\Sigma\left( \theta\right)\left[ J\left( \theta\right) \right]^{T} \right]_{yy}}$ | (A12) |
| --- | --- |

A $\left( 1-\alpha\right)\%$ confidence interval for$P\left( Y_{i}^{*}\geq y \right)$ is then

| $\hat{P}\left( Y_{i}^{*}\geq y \right)\pm z_{\alpha/2}\hat{\mathrm{se}}\left[ \hat{P}\left( Y_{i}^{*}\geq y \right) \right]$ | (A13) |
| --- | --- |

**Multivariate Regression Methodology**

The conditional probabilities of achieving a milestone given successful achievement of the preceding ones was modeled as a logit of a linear combination of study characteristic values. Estimation of the regression coefficients occurred subject to Adaptive Elastic Net constraints (i.e., constraints on the sum of the squares of the regression coefficient estimates and on the sum of the absolute values of the regression coefficients).^1,2^ The number of variables to include and the severity of these constraints were selected by minimizing the Akaike Information Criterion (AIC).^3^ Permutation tests^4^ were used to assess whether the regression coefficient associated with each characteristic was zero.

Let $\theta_{m}\left( x \right)$ be this conditional probability for a study with baseline characteristic values equal to $x$. Then the likelihood function is

| $L\left( \theta\vert x_{i} \right)=\prod_{m=1}^{M} \prod_{i=1}^{N} \left[ 1-\theta_{m}\left( x_{i} \right) \right]^{I\left\{ y_{i}=m-1 \right\}z_{i}}\left[ \theta_{m}\left( x_{i} \right) \right]^{I\left\{ y_{i}\geq m \right\}}$ | (A14) |
| --- | --- |

Assume $\mathrm{logit} \theta_{m}\left( x_{i} \right)=\alpha_{m}+\sum_{j=1}^{p} \beta_{j}x_{ij}$. For $m=1, \ldots, M$, the negative log-likelihood

| $Q\left( \alpha_{1},\ldots, \alpha_{M}, \beta_{1}, \ldots, \beta_{p} \right)=-\sum_{i=1}^{N} I\left\{ y_{i}=m-1 \right\}z_{i}\log\left[ 1-\theta_{m}\left( x_{i} \right) \right]-$  $\sum_{i=1}^{N} I\left\{ y_{i}\geq m \right\}\log\left[ \theta_{m}\left( x_{i} \right) \right]$ | (A15) |
| --- | --- |

is minimized with respect to $\alpha_{m}$ and $\beta_{j}$.

Let $w$ be a vector consisting of the quantities

| $w_{im}=\left\{ \begin{matrix} 1 & \mathrm{if} y_{i}\geq m \\ 0 & \mathrm{if} y_{i}=m-1 \mathrm{and} z_{i}=1 \\ \mathrm{undefined} & \mathrm{otherwise} \end{matrix} \right.$ | (A16) |
| --- | --- |

for all cases and milestones, with case index $i$ varying fastest, and

| $V=\left[ \begin{matrix} I_{M \times M}\otimes1_{N} & I_{M \times M}\otimes X \end{matrix} \right]$ | (A17) |
| --- | --- |

where $X$ is the matrix whose $i^{th}$ row and $j^{th}$ column equals $x_{ij}$, $I_{M \times M}$ is the $M\times M$ identity matrix, $1_{N}$ is a vector of ones of length $N$, and $\otimes$ denotes the Kronecker product. Then minimizing (A15) with respect to $\alpha_{m}$ and $\beta_{j}$ can be done through logistic regression of the vector $\tilde{w}$ with respect to $\tilde{V}$, where $\tilde{w}$ is the vector consisting only of the elements of $w$ for which $w_{im}$ is not undefined and  $\tilde{V}$ is a matrix consisting of the rows of $V$corresponding to these elements of $w$.

To perform variable selection and to stabilize the estimates of $\alpha_{m}$ and $\beta_{j}$, this optimization will occur subject to Adaptive Elastic Net penalties on the regression coefficients $\beta_{j}$, i.e.

| $Q\left( \alpha_{1},\ldots, \alpha_{M}, \beta_{1}, \ldots, \beta_{p} \right)+\lambda_{1}\sum_{j=1}^{p} \omega_{j}\left\vert\beta_{j} \right\vert+\lambda_{2}\sum_{j=1}^{p} \beta_{j}^{2}$ | (A18) |
| --- | --- |

where $\lambda_{1}\geq0$ and $\lambda_{2}\geq0$ are tuning parameters controlling the severity of each term of the penalty and the $\omega_{j}$ are weights controlling the relative severity of the $L_{1}$-norm penalty term for each regression coefficient. $\omega_{j}$ will be set equal to

| $\omega_{j}=\frac{1}{\tilde{\beta}_{j}^{2}}$ | (A19) |
| --- | --- |

where $\tilde{\beta}_{j}$ are ridge regression estimates of the coefficients^5^ using a very small value for the tuning parameter. Both $\lambda_{1}$ and $\lambda_{2}$ will be selected by minimizing the Akaike information criterion

| $2Q\left( \hat{\alpha}_{1},\ldots, \hat{\alpha}_{M}, \hat{\beta}_{1}, \ldots, \hat{\beta}_{p} \right)-2\nu\left( \lambda_{1}, \lambda_{2} \right)$ | (A20) |
| --- | --- |

where

| $\nu\left( \lambda_{1}, \lambda_{2} \right)=\mathrm{tr}\left\{ \tilde{V}_{J}\left( \tilde{V}_{J}^{T}\tilde{V}_{J}+\lambda_{2}\left[ \begin{matrix} 0_{M\times M} & 0_{M\times\left\vert J \right\vert} \\ 0_{\left\vert J \right\vert\times M} & I_{\left\vert J \right\vert\times\left\vert J \right\vert} \end{matrix} \right] \right)^{-1}\tilde{V}_{J}^{T} \right\}$ | (A21) |
| --- | --- |

is the effective degrees of freedom given specified values of $\lambda_{1}$ and $\lambda_{2}$ and $J$ is the set of indices for which  $\hat{\beta}_{j}\neq0$. $0_{P_{1}\times P_{2}}$ is a $P_{1}\times P_{2}$ matrix of zeros and $\mathrm{tr}$ denotes the trace of a matrix.

p-values of the null hypotheses $\beta_{j}=0$ for $j=1,\ldots,p$ will be obtained through a permutation test. Let  $\hat{\beta}_{1},\ldots, \hat{\beta}_{p}$ denote the estimates of $\beta_{1},\ldots, \beta_{p}$ given the observed data. The indices on $\left( y_{1},z_{1} \right), \left( y_{2},z_{2} \right), \ldots, \left( y_{N}, z_{N} \right)$ will be permuted to produce simulations of the outcome variables under the null distribution $y_{1}^{'}, y_{2}^{'},\ldots, y_{N}^{'}$ and $z_{1}^{'}, z_{2}^{'}, \ldots, z_{N}^{'}$. The techniques described above will be applied to the $y_{i}^{'}$, $z_{i}^{'}$, and $x_{ij}$ to obtain estimates of the $\beta_{j}$ under the null distribution. This process will be repeated $S=1000$ times to obtain $S$ simulations of  $\hat{\beta}_{j}$ under its null distribution,  $\hat{\beta}_{j}^{\left( 1 \right)}, \hat{\beta}_{j}^{\left( 2 \right)}, \ldots, \hat{\beta}_{j}^{\left( S \right)}$. The p-value is then

| $\frac{1}{S}\sum_{s=1}^{S} I\left\{ \left\vert\hat{\beta}_{j}^{\left( s \right)} \right\vert\geq\left\vert\hat{\beta}_{j} \right\vert\right\}$ | (A22) |
| --- | --- |

**References**

1. Zou HH, T. Regularization and variable selection via the elastic net. *Journal of the Royal Statistical Society Series B* 2005;67(2):301-320.

2. Zou H, Zhang HH. On the Adaptive Elastic-Net with a Diverging Number of Parameters. *Ann Stat.* 2009;37(4):1733-1751.

3. Akaike H. A new look at the statistical model identification. *Institute of Electrical and Electronics Engineers (IEEE) Transactions on Automatic Control* 1974;19(6):716-723.

4. Welch WJ (1990). “Construction of permutation tests”. Journal of American Statistical Association 85, pg. 693-698. .

5. Hoerl AEK, R.W. Ridge regression: Biased estimation for nonorthogonal problems. *Technometrics.* 1970;12(1):55-67.

| **Phase 1 study characteristic** | **Regression coefficient estimate** | **Permutation test p-value** |
| --- | --- | --- |
| Clinical promise observed in phase 1 | $\boldsymbol{1.690}$ | $\boldsymbol{6.00\times1}\boldsymbol{0}^{\boldsymbol{-4}}$ |
| Rationale for study of combination based on in vitro evidence of greater activity of the combination | $\boldsymbol{-1.634}$ | $\boldsymbol{1.80\times1}\boldsymbol{0}^{\boldsymbol{-3}}$ |
| Response biomarker-driven objectives included in phase 1 trial | $\boldsymbol{1.761}$ | $\boldsymbol{2.50\times1}\boldsymbol{0}^{\boldsymbol{-3}}$ |
| Characterizing PK is criterion for phase 1 success | $\boldsymbol{-2.323}$ | $\boldsymbol{2.70\times1}\boldsymbol{0}^{\boldsymbol{-3}}$ |
| Other results observed in phase 1 | $\boldsymbol{-1.893}$ | $\boldsymbol{8.30\times1}\boldsymbol{0}^{\boldsymbol{-3}}$ |
| PK interactions expected | $\boldsymbol{-1.602}$ | $\boldsymbol{0.012}$ |
| Overlapping dose-limiting toxicities expected | $\boldsymbol{-0.970}$ | $\boldsymbol{0.015}$ |
| Rationale for study of combination based on lack of overlapping toxicities | $-0.908$ | $0.021$ |
| 3 + 3 design used for phase 1 | $-1.007$ | $0.028$ |
| Clinical data used for pharmacological or biological rationale for study of the combination | $-0.711$ | $0.036$ |
| Adverse events expected | $0.673$ | $0.040$ |
| Rationale for study of combination based on in vivo evidence of greater activity of the combination | $0.586$ | $0.044$ |
| PK observed in phase 1 | $0.727$ | $0.046$ |
| Optimal dose or schedule established in phase 1 | $0.772$ | $0.055$ |
| Safe and tolerable dose or schedule established in phase 1 | $0.726$ | $0.061$ |
| PD biomarker-driven objectives included in phase 1 trial | $-0.352$ | $0.089$ |

**Supplemental Table 1: Regression coefficient estimates and permutation test p-values of the multivariate model of probability of achieving each milestone given the phase 1 study characteristics.** Characteristics not selected through forward stepwise regression were not included in this table.

**The Survey**

**When answering the following questions, please consider your phase 1 clinical trial [insert NCT number and name]**

**First Non Survey Question (aids in survey completion)**

**Please list collaborators in this trial who would be able to answer questions about the design of the trial.**

Control for adding names and emails of collaborators

**Survey questions**

**1a. Was this trial Investigator Initiated?**

- **Yes**
- **No**

**1b. Who was the primary funder of this trial?**

- **NCI**
- **Industry**
- **Institution**
- **Philanthropic organization**
- **Other**

**Trial Progression Questions [populate references to trial phase with correct trial phase]**

**2. What *a priori* criteria did you use to determine** **the success/failure for your Phase 1 trial (i.e., the criteria defined in the protocol as to whether or not you would move forward to Phase 2 or cease development)? (check all that apply)**

- Establish a safe and tolerable dose or schedule
- Establish an optimal dose or schedule
- Determine the sequence of drug administration
- Show protocol specified pharmacodynamic effect
- Characterize the pharmacokinetics of the drug
- Determine or establish preliminary evidence of activity
- None / not enough information at the time
- Other ____

**3. What were the results of your Phase 1 trial? (check all that apply) [will be populated with criteria selected in question 2]**

- Established a safe and tolerable dose or schedule
  (from Establish a safe and tolerable dose or schedule)
- Established a dose or schedule that was optimal
  (from establish an optimal dose or schedule)
- Determined an optimal sequence of drug administration
  (from determine the sequence of drug administration)
- Observed a preplanned pharmacodynamic effect
  (from show protocol specified pharmacodynamic effect)
- Observed the pharmacokinetics of the drug
  (from characterize the pharmacokinetics of the drug)
- Observed a pharmacodynamic effect
  (from determine or establish preliminary evidence of activity)
- Observed Clinical Promise
- Other ______
- None

**4. The following question seeks data on how far the combination that was tested in your Phase 1 trial has progressed toward regulatory approval.**

**Please select the category that best describes your trial and its combination. (check one)**

- No further study of the combination was performed due to toxicity or lack of response
- No further study of the combination was performed due to non-clinical reasons (for example lack of financing from drug company)
- Further study of the combination was warranted, but the subsequent Phase 2 trial did not occur
- Further study of the combination was warranted and a subsequent trial was developed or is in development

[If this last item is checked, the following questions pop up]

**4a. What is the highest phase of study reached for this combination? (check one)**

- - Phase 2 (includes phase 2 portion of a phase 2/3 trial)
  - Phase 3 (includes phase 3 portion of a phase 2/3 trial)

**4b. Which statement below best describes the status of the trial indicated in question 4a? (check one)**

- - - Combination received regulatory approval
    - Study is in development
    - Study is currently ongoing
    - Study was activated but ceased prior to completion
    - Study was activated but is temporarily on hold / closed
    - Other ____
    - Study is completed

[If the last one is checked, then the following questions pops up:]

**4b1. Did the trial indicated in 4a meet its predefined primary endpoint?**

- - - - No
      - Yes
      - Results are still pending

**4b2. Did the trial indicated in 4a meet its predefined secondary endpoint(s)?**

- - - - No
      - Yes
      - Results are still pending

**4b3.** **Were the results of the trial indicated in 4a published?**

- - - - No
      - Yes, as an abstract
      - Yes, in paper form
      - Results are still pending

**5. Which best describes the combination’s approval status for your Phase 1 trial? (check one)**

- The combination has not been approved by the FDA
- The combination was approved by the FDA after phase 1
- The combination was approved by the FDA after phase 2
- The combination was approved by the FDA after phase 3

**Trial Design Questions**

**Preclinical data questions on the rationale of your trial:**

**6. Which type of data was used for the pharmacological or biological rationale of your trial?** **(Check all that apply)**

- Preclinical *in vitro* (cell)
- Preclinical *in vivo* (animal)
- Clinical data
- Other _____
- Not sure/Don’t know

**6a. [Question asked if 6 checked *'in vitro*'] What preclinical *in vitro* factors supported the combination tested in your trial?** **(check one):**

- - Evidence of greater activity of the combination compared with the activity of either agent alone in *in vitro* nonclinical models
  - No evidence of greater or less activity of the combination compared with the activity of either agent *vitro* alone in *in vitro* nonclinical models.
  - Evidence of less activity of the combination compared with the activity of either agent alone in *in vitro* nonclinical models.

**6a1. [Question asked if 6 checked *'in vivo*'] What preclinical *in vivo* factors supported the combination tested in your trial?** **(check all):**

- - Evidence of greater activity of the combination compared with the activity of either agent alone in *in vivo* nonclinical models
  - No evidence of greater or less activity of the combination compared with the activity of either agent alone in *in vivo* nonclinical models.
  - Evidence of less activity of the combination compared with the activity of either agent alone in *in vivo* nonclinical models.
  - Lack of evidence supporting overlapping toxicities.

**7. Did you have any primary or secondary biomarker-driven objectives in your trial?**

- No
- Yes

[If yes, the following pops up]

**What types of biomarkers? (check all that apply):**

- - Pharmacodynamic (effect relative to a targeted defect or pathway)
  - Predictive (does the biomarker explain differences in treatment effect)
  - Prognostic (likely outcome of the patient under standard therapy)
  - Response (to assess changes in the biomarker following treatment; may include imaging biomarkers)
  - Exploratory
  - Other _______

**8. When combining 2 drugs, sometimes there are potentially negative drug-drug interactions. How much of a concern was this in your Phase 1 trial?**

- Was not a concern
- Somewhat concerned
- Very concerned

[If 'Somewhat concerned' or 'Very concerned']

**8a. Did you test for this concern in your Phase 1 trial?**

- - Yes
  - No

**9. Did you anticipate potential overlapping dose-limiting toxicities (DLTs) in your Phase 1 study?**

- No
- Yes

[If yes, the following pops up]

**Please describe how you incorporated the interaction into the design of your study: _______**

**10. How familiar are you with the 2014 Clinical Design Task Force’s recommended process for the determination of the Phase 1 combination trial design (see diagram below)?**

- Not at all familiar
- Somewhat familiar
- Very familiar


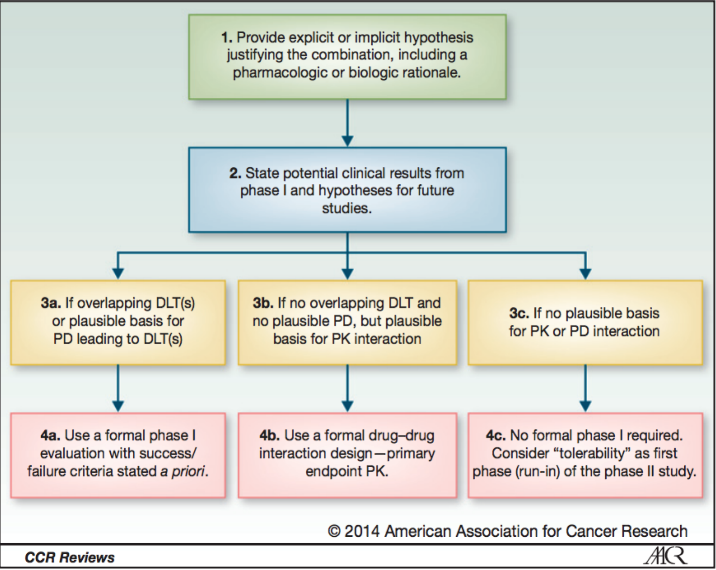


**11. Please point us to the publications or other documents where we can find published results of your trial and preclinical data on the combination.** [possible publications will be automatically identified from user name, trial identifier and Pubmed], answers will be in the form of Pubmed IDs.

II. Search terms used to identify trials for inclusion:

Clinical trials were selected that used drugs from the below table. Each line gives a drug name along with the searched synonyms.

| 90Y-cG250  abemaciclib,LY2835219  abiraterone acetate,Zytiga  ABT-510  Acalisib,CAL-120,GS-9820  ACE-041,Dalantercept  adecatumumab  ado-trastuzumab emtansine,Kadcyla  AEE788  AEG35156,GEM640  afatinib dimaleate,Gilotrif  aflibercept,Zaltrap,Ziv-Aflibercept,VEGF-Trap  AFP464  AGRO 100,AS1411  AGS-PSCA  ALB 109564  alectinib,ROS5424802,AF802,CH5424802  alemtuzumab,Campath  Alisertib,MLN-8237  Alitretinoin,Panretin  alvespimycin,17-DMAG  amatuximab,MORab-009  AMG337  AMG880  anastrozole,Arimidex  AP12009  AP2611  APZ24534  ARRY-380,ONT-380  ARRY520  ASP3026  AT7519M  axitinib,Inlyta,AG013736  Azacitidine,ladakamycin  AZD1775,MK-1775  AZD2281,Olaparib,KU-0059436  AZD4547  AZD4877  AZD6126,ANG453  AZD8055  AZD8330,ARRY-424704,ARRY-704  AZD9291  bafetinib,NS-187,INNO-406  barasertib,AZD1152  BAY 86-9766,refametinib  BAY1000394  BAY806946  belinostat,Beleodaq  beloranib,CKD-732  Bevacizumab,Avastin  bexarotene,Targretin  BEZ235  BGJ398  BHQ880  BI 2536  BI 811283  BIBF120  BIIB021,CNF2024  Birinapant,TL32711  BKM120,buparlisib  blinatumomab,Blincyto,AMG-103,MT-103  BMN673  BMS-214662  BMS-690514  BMS-754807  BMS-833923,XL-139  BMS936559  bortezomib,Velcade,PS-341  bosutinib,Bosulif  brentuximab vedotin,Adcetris,SGN-35  Brivnaib,BMS582664  BYL719,Alpelisib  Cabozantinib,Cometriq,XL-184  canertinib,CI-1033  carfilzomib,Kyprolis  CC223  CDX-1401  Cediranib,AZD2171,Recentin  Cenisertib,AS703569  ceritinib,LDK378,Zykadia  Cetuximab,Erbitux  CF102  cG250-Lu177  chidamide  CHR3996  CHS 828  CI-1040  rocilinostat,ACY1215  romidepsin,Istodax,Depsipeptide,FK228  RTA 402,Bardoxolone methyl  rucaparib,AG14699  ruxolitinib phosphate,Jakafi  S4014813  salirasib  SAM486A,CGP48664  SAR245409,XL765  saracatinib,AZD0530  SB2312  SB743921  SB939,pracinostat  SCH 66336  SCH 900105,AV299  quisinostat,JNJ26481585  R1507  R306405  R547  RAF265,CHIR265  ramucirumab,Cyramza,IMC-1121B  RAV12  Recombinant Human Interleukin-15  regorafenib,Stivarga  Reolysin  resminostat,BYK408740  retaspimycin,IPI504  RG7446,MPDL3280A  RGB 286638  ribociclib,Lee011 | CIGB 300  Cixutumumab,IMC-A12  CO-1686  cobimetinib,GDC0973,XL518  combretastatin  conatumumab,AMG 655  CP-609,754  CP-724,714  CP-870,893  CPI-613  Crenolanib,CP-868,596  crizotinib,Xalkori,PF-02341066  crolibulin,EPC2407  CS-7017  CT-2584 HMS  CTCE-9908  CUDC-101  CVX-045  CX-5461  CXR1002  D-3263  dabrafenib,Tafinlar,GSK2118436B  dacomitinib,PF-00299804  dalotuzumab,MK-0646  danusertib  daratumumab  dasatinib,Sprycel,BMS-354825  decitabine  deforolimus  denileukin diftitox,Ontak  Denosumab,Xgeva  DENSPM  diazepinomicin,TLN-4601,ECO 4601,BU 4664L,ECO 04601  Dinutuximab,Unituxin  Dovitinib,TKI258  DS3078a  E6201  E7070  E7820  Elisidepsin,PM02734  elotuzumab  endostatin  Entinostat,MS-275,SNDX-275  ENZ-2208  enzalutamide,Xtandi,MDV3100  enzastaurin  Eribulin,Halavin,E7389  Erismodegib,LDE225,sonidegib  erlotinib,Tarceva,OSI-774  ertumaxomab  everolimus,Afinitor,RAD001  exemestane,Aromasin  EZN-2968,SPC2968  farletuzumab,MORAb003  FAU  Figitumumab,CP-751,871,okadaic acid  flavopiridol  Foretinib,GSK1363089,XL880  FT-101  fulvestrant,Faslodex  ganetespib,STA9090  Ganitumab,AMG-479  GDC-0152  GDC0980  gefitinib,Iressa  GEM231  Girentuximab,cG250  Glembatumumab vedotin,CDX-011  GSAO  GSK2126458  GSK2141795  GSK461364  GSK923295A  GTI-2040  HGS-TR2J,KMTR2,HGS-ETR2  HGS1036,FP-1039,GSK3052230  HMN-214,IVX-214  Hu3s193,Rebmab 100  HuMV833  Ibritumomab tiuxetan,Zevalin  ibrutinib,Imbruvica,PCI-32765  Icotinib  icrucumab,IMC-18F1  idelalisib,Zydelig  Imatinib mesylate,Gleevec  IMC-1C11  imetelstat,GRN163L  Imexon  imgatuzumab,RO4987655,GA201,RG7160  XL999  XMT-1001  YM 155,sepantronium bromide  YM753,OBP801  ZK CDK,ZK 304709  SLS-314  SNX-5422,PF-04929113  Sonepcizumab  sorafenib,Nexavar,BAY 43-9006,tosylate,BAY 54-9085  squalamine  SU014813  sunitinib,Sutent,SU011248  T-dCyd  Tacedinaline,Acetyldinaline,CI994  TAK-285  Talazoparib,BMN 673  tamoxifen  Tanespimycin,17-AAG  seliciclib  Selumetinib,AZD6244  SF1126  SGI-110,S110  SGI-1252  SGX126  sibrotuzumab  Silmitasertib,CX-4945  siltuximab,Sylvant | INC280  INCB024360  iniparib,BSI201  Intetumumab,CNTO 95  IPdR  IPI-926,saridegib  Ipilimumab,Yervoy,MDX-010  ISIS 2503  ISIS 5132,CGP 69846A  isosorbide,AT-101,Devicoran  Ixazomib,MLN9708  JNJ-26854165,serdemetan  JQ1  KML001  KRN5500  KU-0063794  KX2-391,KX01  L-778123  Lanreotide acetate,Somatuline  lapatinib,Tykerb,GW572016  LAQ824,dacinostat  LDY135  LEE001  lenalidomide,Revlimid,CC-5013  lenvatinib mesylate,Lenvima  LErafAON  letrozole,Femara  lexatumumab  lexibulin,CYT997  linifanib,ABT-869  Linsitinib,OSI-906  Lorvotuzumab mertansine,IMGN901  Luminespib,AUY922  LY2157299,galunisertib  LY2181308  mapatumumab  Marizomib,NPI0052,Salinosporamide  masitinib  matuzumab,EMD72000  MB07133  MDX1203  MDX1411  MEDI-575  MEDI4736  metastat  Methoxyamine hydrochloride,TRC-102  MG98  MGA33  MGCD265  MK-2461  MK-5108,VX-689  MK2206  MK4827,nirapirab  MLN0128,INK128  MLN4924  MLN8054  MM-111  MM-121,SAR256212  MMI270  MNRP1685A  Mocetinostat,MGCD0103  Motesanib diphosphate,AMG706  MP470,Amuvatinib  Naptumomab estafenatox,ABR-217620  Navitoclax,ABT263  Necitumumab,IMC-11F8  Neratinib  NGR-hTNF  nilotinib,Tasigna  Nimozutumab  nintedanib,BIBF1120,OFEV  nivolumab,Opdivo,MDX1106,OO4538,BMS-936558  NMS-1116354  NPI-2350,phenylahistin  NPI-2358,plinabulin  NSC 686288,aminoflavone  NV-196,triphendiol,AB196  NVP-BEZ235,dactolisib  obatoclax  obinutuzumab,Gazyva  Oblimersen  OBP-301,Telomelysin  ofatumumab,Arzerra  OGX 427,apatorsen  OGX-011,custirsen  olaparib,Lynparza  ombrabulin,AVE8062  ON 01910.Na  Onalespib lactate,AT13387  TRC-105  Trebananib,AMG386  tremelimumab  Tretinoin,Vesanoid  Triapine  TSU-68,Orantinib  UCN-01  UNBS5162  UNII-8P77G99D3P,ANG1005  Valproic acid  vandetanib,Caprelsa,ZD6474  vantictumab,OMP18R5  varlitinib,ARRY-334543  vatalanib,PTK787  Veliparib,ABT-888  vemurafenib,Zelboraf  Vismodegib,Erivedge,GDC-0449  Volasertib,BI 6727  volociximab  voreloxin,SNS595,vasaroxin,AG7352  vorinostat,Zolinza,SAHA  Vorsetuzumab mafodotin,SGN75  VX-970  X-396  tasquinimod  Tefinostat,CHR2845  teglarinad,GMX1777  telatinib  temsirolimus,Torisel,CCI-779  TH-302,Evofosfamide  tigatuzumab,CS-1008  tipifarnib  Tivantinib,ARQ-197  tivozanib,AV-951  TLC388,Lipotecan | Onartuzumab,MetMab  Oprozomib,ONX0912  ornatuzumab  OSI 930  OXA-01  P276-00  palbociclib,Ibrance,PD0332991  panitumumab,Vectibix  panobinostat,Farydak,LBH589  pazopanib,Votrient,GW786034  PCI-24781,abexinostat  PD-0325901  pegdinetanib,CT-322  pelitinib,EKB-569  pembrolizumab,Keytruda,MK-3475,lambrolizumab  perifosine  pertuzumab,Perjeta  PF-00562271  PF-03732010  PF-04691502  PF-05212384,PKI587  PF-3758309  PF00299804  PHA-848125,milciclib  phenylbutyrate  PHL 050  PHT-427  picropodophyllotoxin,AXL1717,picropodophyllin  Pictilisib,GDC0941  pimasertib,AS703026,MSC1936369B  pivanex,AN-9  PKC412,midostaurin  PKI166  plerixafor,AMD3100  PNU-145156E  pomalidomide,Pomalyst,CC-4047  pralatrexate,Folotyn  PRLX 93936  PRO95780  PTX-008  PX-12  PX866  QBI-139  tozasertib,MK-0457  trametinib,Mekinist,GSK112021,MEK162  Trastuzumab,Herceptin  XL228  XL647,Tesevatinib,EXEL-7647,KD-019  SCH727965,dinaciclib  rituximab,Rituxan  Ro 31-7453  RO4929097  RO5083945  toremifene,Fareston  tosedosta  Sirolimus,rapamune  XL147  ridaforolimus  Rilotumumab,AMG102 |
| --- | --- | --- | --- |

Trials with conditions matching all or part of items from the below list were selected:

- Metastatic
- Cancer
- Myeloma
- ALL
- MDS
- lukem
- leukem
- carcinoma
- leuc
- SCLC
- AML
- neoplasm
- lymphoma
- CML
- myelodysp
- HNSCC
- sarcoma
- malign
- DLBCL
- tumor
- GIST
- tumour
- CLL
- myelofibro
- oma

Trials with conditions matching all or part of items from the below list were excluded:

- glaucoma
- gynecomastia
- neurofibroma
- aroma
- autosomal
